# Wilms Tumor 1 Gene Mutations in Patients with Cytogenetically Normal Acute Myeloid Leukemia

**DOI:** 10.4274/tjh.2012.0210

**Published:** 2014-06-10

**Authors:** Salah Aref, Solafa El Sharawy, Mohamed Sabry, Emad Azmy, Dalia Abdel Raouf, Nadia El Menshawy

**Affiliations:** 1 Mansoura University Faculty of Medicine, Department of Clinical Pathology, Hematology Unit, Mansoura, Egypt; 2 Mansoura University Faculty of Medicine, Mansoura Cancer Institute, Clinical Hematology Unit, Mansoura, Egypt

**Keywords:** Acute myeloid leukemia, Cytogenetically normal, Mutations, prognosis, Wilms tumor 1 gene

## Abstract

**Objective:** This study aimed to assess the prognostic impact of Wilms tumor 1 (WT1) mutations in cytogenetically normal acute myeloid leukemia (CN-AML) among Egyptian patients.

**Materials and Methods:** Exons 1, 2, 3, 7, 8, and 9 of WT1 were screened for mutations in samples from 82 CN-AML patients out of 203 newly diagnosed AML patients, of age ranging from 21 to 74 years, using high-resolution capillary electrophoresis.

**Results:** Eleven patients out of 82 (13.41%) harbored WT1 mutations. Mutations were detected in exon 7 (n=7), exon 9 (n=2), exon 8 (n=1), and exon 3 (n=1), but not in exons 1 or 2. There was no statistically significant difference between the WT1 mutants and wild types as regards age, sex, French-American-British subtypes, and the prevalence of success of induction remission therapy (p=0.966; 28.6% vs. 29.3%). Patients with WT1 mutations had overall survival lower than patients with the wild type (HR=1.38; 95% CI 4.79-6.86; p=0.004).

**Conclusion:** CN-AML patients with WT1 mutations have poor clinical outcome. We recommend molecular testing for WT1 mutations in patients with CN-AML at diagnosis in order to improve risk stratification of those patients.

## Abstract (tr)

**Amaç:** Bu çalışma sitogenetiği normal akut miyeloid lösemi (SN-AML) tanısı olan Mısırlı hastalarda Wilms tümör 1 (WT1) mutasyonlarının prognoz üzerine etkilerini incelemeyi amaçlamaktadır.

**Gereç ve Yöntemler:** İki yüz üç yeni tanılı AML hastası arasından yaşları 21 ile 74 arasında değişen 82 SN-AML hastasına ait örneklerde exon 1, 2, 3, 7, 8 ve 9 WT1’e ait mutasyonlar açısından yüksek çözünürlüklü kapiller elektroforez kullanılarak tarandı.

**Bulgular:** Seksen iki hastanın 11’i (%13,41) WT1 mutasyonlarını taşımaktaydı. Mutasyonlar exon 7 (n=7), exon 9 (n=2), exon 8 (n=1) ve exon 3’te (n=1) tespit edildi, ancak exon 1 veya 2’de tespit edilmedi. WT1 mutasyonunu taşıyanlar ile taşımayanlar arasında yaş, cinsiyet, French-American-British (FAB) alt tipleri ve indüksiyon tedavisindeki başarı açısından istatistiksel anlamlı farklılık saptanmadı (p=0.966; 2%8.6 vs. %29.3). WT1 mutasyonu olan hastalarda genel sağkalım mutasyonu olmayanlara göre daha düşük bulundu (HR=1.38; %95 CI 4.79-6.86; p=0.004).

**Sonuç:** WT1 mutasyonu olan SN-AML hastalarında klinik gidiş kötüdür. SN-AML hastalarında tanı anında daha iyi bir risk sınıflaması yapmak için WT1 mutasyonlarının moleküler testlerle araştırılmasını önermekteyiz.

## INTRODUCTION

Acute myeloid leukemia (AML) is a clinically and genetically heterogeneous disease that accounts for 20% and 70% of acute leukemia in children and adults, respectively. The cytogenetic finding is considered as the cardinal marker for AML risk stratification. Cytogenetically normal AML (CN-AML) is a large cytogenetic subgroup of AML, representing approximately 45% of adult patients with AML who are younger than 60 years. During the last decade, the prognostic stratification of CN-AML was based on several molecular markers including the nucleophosmin 1 gene, the fms-related tyrosine kinase 3 gene, the CCAAT/enhancer-binding protein alpha gene, the myeloid-lymphoid or mixed-lineage leukemia gene, the neuroblastoma RAS viral oncogene homolog gene, the runt-related transcription factor 1 gene, and the Wilms tumor 1 gene (WT1) [1,2].

WT1 was identified as a tumor suppressor gene, located at chromosome 11p and encoding a transcription factor with an N-terminal transcriptional regulatory domain (exons 1 to 6) and a C-terminal 4-Cys2His2 zinc finger domain (exons 7 to 10) [3,4].

WT1 expression occurs primarily in cells of the developing genitourinary and hematopoietic systems and is inversely correlated with the degree of differentiation in both systems. In hematopoiesis, expression occurs in CD34 progenitor cells but is absent in mature leukocytes. The precise role of WT1 in normal and malignant hematopoiesis remains controversial [5,6]. It has been implicated in regulation of cell survival, proliferation, and differentiation, and it may function as an oncogene. However, this diversity may reflect both tissue specificity of downstream targets and expression of different isoforms, given that alternatively spliced isoforms and post-transcriptional modifications are thought to control the cellular and functional properties of the protein [7,8].

WT1 mutations have been found in about 10%-15% of cases of AML and 20% of cases of biphenotypic leukemia, but mutations are rare in acute lymphoblastic leukemia (ALL) [2]. In AML, the WT1 mutations cluster mainly in exons 7 and 9, and less frequently in exons 1, 2, 3, and 8 [9,10].

The aim of this study was to evaluate the incidence and clinical impact of WT1 mutations in adult patients with CN-AML.

## MATERIALS AND METHODS

**Subjects and Treatment Protocols**

The present study was carried out on 82 adult patients (21-74 years) from the hematology unit of the Mansoura Cancer Institute between June 2009 and January 2011, after patients provided written consent. The diagnosis of AML was based on the presence of blast cells at ≥20% in bone marrow (BM) smears. Eighty patients had de novo AML and 2 had secondary AML (5 M1, 18 M2, 30 M4, 23 M5, 6 M6). The diagnosis and French-American-British subtypes were confirmed by immunophenotyping using a Coulter Epics XL Flowcytometer (PN 42372238 B, Coulter Corporation, Miami, FL, USA) to confirm diagnosis with Cyt. MPO, CD13, CD33, and CD117 as a primary panel for myeloid lineage; CD14, CD36, and CD11b for M4 and M5; CD61 and glycophorin A for M6; and CD41 and CD42 for M7. The patients were observed for 12 months or until death. History taking and clinical examination for organomegaly were done for all patients. 

All patients gave informed consent for both treatment and genetic analysis. All patients received intensive induction therapy (cytarabine, 100 mg/m2/day for 7 days of i. v. continuous infusion, and daunorubicin, 45 mg/m2/day for 3 days i. v.) and consolidation therapy (cytarabine, 3 g/m2/12 h for 3 days repeated for 3-6 cycles). 

**Methods**

**Cytogenetic and Molecular Genetic Analysis**

Pretreatment blood samples from all patients were studied by chromosome banding analysis to improve the accuracy of cytogenetic diagnosis and to exclude cytogenetically abnormal AML. The specimens were also analyzed by fluorescence in situ hybridization for the presence of t (8;21) (q22;q22) for M2, t (15;17) (q22;q12) for M3, inv (16) (p13q22) for M4E, or 11q23 for M5.

**Determination of Mutation Status in CN-AML**

WT1 exons 1, 2, 3, 7, 8, and 9 were amplified using polymerase chain reaction (PCR) with approximately 50 ng of genomic DNA, 1X QIAGEN Multiplex PCR Master Mix, and 10 pmol of primers (6` FAM end-labeled forward primers) designed to flank intronic regions. For exon 1, due to the long size and some difficulties in fragment amplification, 3 different primer pairs were used: 1F (5`-AGCCAGAGCAGCAGGGAGT -3`) and 1R (5`- ACGACCCGTAAGCCGAAGC -3`), annealing temperature was 64 °C; 1F (5`- ATGGGCTCCGACGTGC -3`) and 1R (5`- ATGAAGGAGTGAGGCGG -3`), annealing temperature was 54 °C; 1F (5`- TTCGGCTTACGGGTCGTTGG -3`) and 1R (5`- CAAAAGGGGTAGGAGAGGGG -3`), annealing temperature was 62 °C. For exon 2, 2F (5`- CCGTCTTGCGAGAGCACC -3`) and 2R (5`- CTAATTTGCTGTGGGTTAGG -3`) were used with an annealing temperature of 58 °C; for exon 3, 3F (5`- GCTCAGGATCTCGTGTCTCC -3`) and 3R (5`- GCCTCCAAGACCCAGCAT -3`), annealing temperature was 64 °C; for exon 7, 7F (5`- GACCTACGTGAATGTTCACATG -3`) and 7R (5`- ACCAACA CCTGGATCAGACCT -3`), annealing temperature was 60 °C; for exon 8, 8F (5`- GAGATCCCCTTTTCCAG -3`) and 8R (5`- CACAGCTGCCAGCAATG -3`), annealing temperature was 56 °C; and for exon 9, 9F (5′- CTCACTGTGCCCACATTG -3′) and 9R 5’- CAATTTCATTCCACAATAG -3’), annealing temperature was 58 °C (Applied Biosystems, Foster City, CA, USA). Thirty-five cycles of amplification were performed. PCR products were then analyzed by fragment analysis using high-resolution capillary electrophoresis on an ABI 310 Genetic Analyzer (PE Applied Biosystems, Foster City, CA, USA). The wild-type amplicon was 348 bp. For samples with additional peaks representing mutant amplicons, the relative mutant level was calculated from the area under the curve and expressed as a percentage of total WT1 alleles (mutant/[wild type + mutant(s)] x 100). 

**Statistical**

The statistical analysis of data was done using Excel and SPSS 16. Qualitative data were described in the form of numbers and percentages. Quantitative data were described in the form of mean ± standard deviation (SD). Statistical analysis was done by comparison between groups using the chi-square test regarding qualitative data, while quantitative nonparametric data comparison was performed using one-way ANOVA and the paired samples t-test. The probability of results being explained by chance (p-value) was calculated for all parameters (significance at p≤0.05 with 95% confidence interval). Overall survival (OS) was defined as the time from diagnosis to the last follow-up or death from any cause. The probabilities of OS were estimated by the Kaplan-Meier method, and differences between 2 survival distributions were compared with the log-rank test.

## DISCUSSION

**Prevalence of WT1 Mutations **

**Figure 1:**
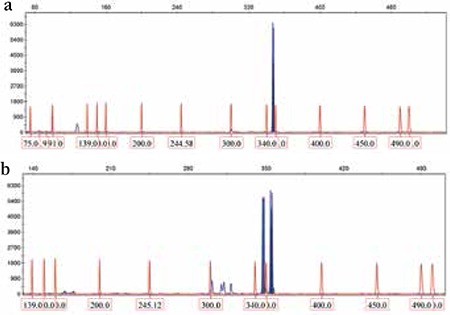
Capillary electrophoresis of WT1 exon 7: a) wild-type peak at 348 bp; B) wild-type peak at 348 bp and mutant peaks at 355 bp.

WT1 mutations were identified in 11 out of 82 (13.41%) patients. Mutations were detected in exon 7 (n=7), exon 9 (n=2), exon 3 (n=1), and exon 8 (n=1) but were not detected in exons 1 and 2. Detection was performed on diagnostic DNA samples from 82 adult CN-AML patients by using high-resolution capillary electrophoresis. The mutation levels ranged from 6% to 51% in the form of additional peaks at 355, 360, 373, and 390 bp (Figure 1).

**Patient Characteristics in Relation to WT1 Mutation Status**

Characteristics of the WT1 mutant and wild-type cases are shown in Table 1. There was no difference between the 2 groups in terms of age, sex, type of leukemia (de novo or secondary), French-American-British subtypes, and complete blood count at presentation. However, there was a significant difference between the 2 groups regarding the bone marrow blast cell percentage (p=0.038).

**Response to Therapy and Clinical Outcome in WT1 Mutant-Positive Patients**

**Figure 2:**
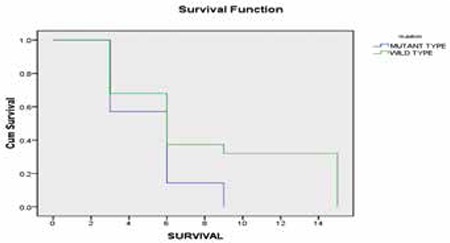
Overall survival in CN-AML patients according to the mutational status of WT1. The overall survival time was significantly shorter in the group of CN-AML patients harboring WT1 mutations as compared to those with the wild type (p=0.004).

In univariate analysis, there was no difference in the rates of complete remission (CR) (18.2% vs. 49.2%) between WT1-mutant and WT1 wild-type patients, respectively (Table 2). On the other hand, the patients with WT1 mutations had shorter OS than patients with wild-type WT1 (HR=1.38; 95% CI 4.79-6.86; p=0.004). The percentage of living wild-type patients was 37.3% versus 14.2% in the mutant-type patients at 6 months and 32% versus 0.0% at 12 months, respectively (Figure 2).

## RESULTS

In a previous study of AML, the WT1 mutations predominantly clustered in exon 7 (mostly frameshift mutations resulting from insertions or deletions) and less frequently in exon 9 (mostly substitutions), whereas in patients with Denys-Drash syndrome or Frasier syndrome, the majority of WT1 mutations were point mutations located either in Zn fingers 2 and 3 (exons 8 and 9) or, in cases of Frasier syndrome, in intron 9 [2].

WT1 mutations were identified in 11 cases out of the 82 adult CN-AML patients (13.41%) in the present study. This figure is slightly higher than those reported by Virappane et al. [8] of 10% and by Paschka et al. [11] of 10.7%, but is in accordance with that reported by Gaidzik et al. [2]. The difference may be explained by the fact that, in our study, the entire WT1 coding sequence was analyzed, and mutations other than those in exons 7 or 9 were identified.

In the current study, there was no significant difference between the WT1 mutant and wild type as regards age, sex, WBC count, hemoglobin concentration, platelet count, and French-American-British subtypes. Similar findings were reported by Virappane et al. [8]. On the other hand, Paschka et al. [11] and Gaidzik et al. [2] demonstrated a significant difference between the WT1 mutant and wild type as regards WBC counts.

In our study, a significant association between the WT1 mutation and high BM blast counts was detected. This finding is in agreement with those of Virappane et al. [8], Paschka et al. [11], and Gaidzik et al. [2], who stated that the WT1 mutation might induce high proliferation capacity of blast cells.

The results in this study revealed that there was a non-significant difference between the WT1 mutant and wild type in rate of induction remission (IR). This agrees with the finding of Paschka et al. [11] and Gaidzik et al. [2], who did not find a relation between WT1 mutations and achievement of IR. On the other hand, Virappane et al. [8] found that patients with WT1 mutations had an inferior response to induction chemotherapy, with a low CR rate and higher induction death rate. 

In the present study, CN-AML patients with WT1 mutation had short OS (2.7 months) as compared to patients with the wild type (5.8 months). The difference was statistically significant (p=0.004). This result is in agreement with those of Paschka et al. [11] and Virappane et al. [8], who found that mutations in the WT1 gene were independent predictors for worse disease-free survival and OS in CN-AML. On the other hand, Gaidzik et al. [2] reported no correlation between the presence of WT1 mutations and OS. Moreover, in childhood ALL, Özgen et al. [12] stated that the degree of WT1 expression had no impact on patients’ survival. The effect of WT1 mutation on a patient’s survival could be explained on the basis that patients with mutated WT1 have high expressions of ERG and BAALC more frequently than do patients with unmutated WT1 [13]. Over-expression of both the ERG and the BAALC genes has been associated with an adverse prognosis. Moreover, WT1 mutations would be expected to abolish, impair, or change the DNA-binding ability of the WT1 protein to its target genes, including those that encode proteins involved in the regulation of normal hematopoiesis (RARA, CSF1), apoptosis (BCL2, BCL2A1, BAK1), cell cycle (CCNE1, CDKN1A), gene transcription (MYC, PAX2, MYB, EGR1), and cell proliferation (TGFB1, PDGFA) [11,14].

In conclusion, CN-AML patients with WT1 mutation have poor clinical outcome. We recommend molecular testing for WT1 mutations in patients with CN-AML at diagnosis in order to improve risk stratifications of those patients.

As a limitation of this study, the number of patients enrolled was relatively small, and during statistical analysis we found that there was a need to sub-classify the AML patients into those harboring mutations of WT1 and those with the wild type. This led to a decrease in the statistical power of some analyses. We therefore suggest that future studies include larger groups. 

## CONFLICT OF INTEREST STATEMENT

The authors of this paper have no conflicts of interest, including specific financial interests, relationships, and/or affiliations relevant to the subject matter or materials included. 

## Figures and Tables

**Table 1:**
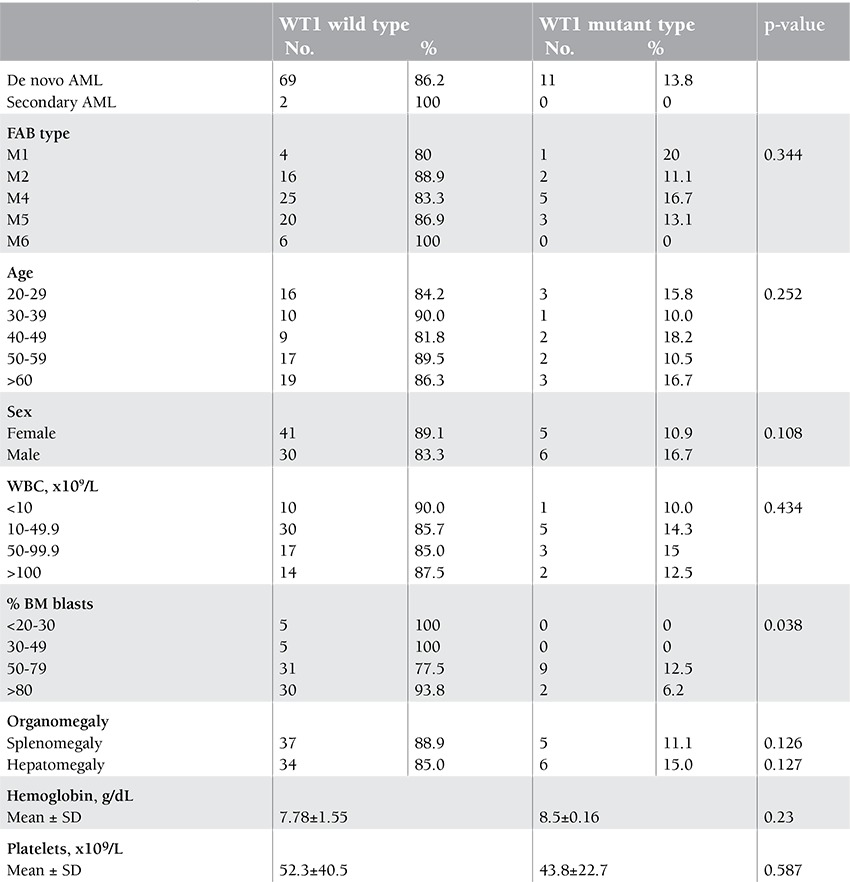
Clinical and demographic characteristics of the 82 CN-AML patients studied.

**Table 2:**
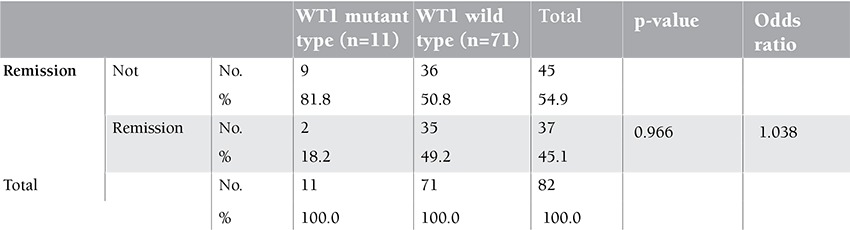
Response to therapy in WT1 mutant and wild-type patients.
